# AMH standardization and preanalytical phase as two sides of the same coin

**DOI:** 10.5935/1518-0557.20220076

**Published:** 2023

**Authors:** Gülşah Demirci, Koray Görkem Saçıntı, Murat Sönmezer, Hasan Serdar Öztürk

**Affiliations:** 1 Department of Medical Biochemistry, Ankara University School of Medicine, Ankara, Turkey; 2 Department of Obstetrics and Gynecology, Ankara University School of Medicine, Ankara, Turkey

Dear Editor,

The primary goal of treatment individualization in *in vitro* fertilization (IVF) is to provide individualized ovarian stimulation, protocol selection, and gonadotrophin starting dose; thereby optimizing oocyte yield, avoiding OHSS, and reducing cycle cancellations (La Marca & Sunkara, 2014). The most reliable ovarian reserve markers should determine the optimal stimulation strategy in IVF. Although numerous biomarkers have been identified as predictors of ovarian response, measurement of circulating anti-Mullerian hormone (AMH) is widely employed to predict ovarian response and determine the dosing algorithm of *in vitro* fertilization treatment, particularly the ability to react to exogenous gonadotropins. Predicting ovarian response also enables more precise communication with patients on the possibility of hazards or cancellation, the likelihood of a good result, and cumulative live birth rates that increase in lockstep with the number of retrieved oocytes.

Immunological methods are mostly preferred for AMH measurement. Currently, since no reference standard material has been developed for AMH measurement, immunoassays are calibrated by independent manufacturer calibrators, which increases the variation between different methods (Punchoo & Bhoora, 2021). Between-method bias values were reported as -25.2% to -9% at 1 ng/mL concentration and 34% to 45% at 5 ng/mL concentration in a study comparing AMH results from different immunoassays (Punchoo & Bhoora, 2021). Since the comparability of results by the clinician decreases as the variation between methods increases, standardization of immunological methods is important for appropriate follow-up of patients from different clinics.

The AMH measurement by Beckman Coulter Access assay was 10% higher than Roche Elecsys assay (Iliodromiti *et al*., 2017). And this difference between the two automated analyzers was found to result in 29% of women being classified with an incorrect follitropin delta dose (Iliodromiti *et al*., 2017). As disagreement between methods may affect clinical decisions, standardization of AMH measurement is also necessary for a standard clinical approach and an accurate dosing algorithm. Distribution of the same International Reference Reagents for accurate calibration of AMH is one step to be taken. Standardization of analyte-specific antibodies is another crucial step for more comparable AMH results. The establishment of working groups including clinicians and laboratory professionals might improve the between-method agreement.

On the other hand, accurate management of the pre-laboratory period and sample collection also needs to be addressed for more clinically comparable results. Sample collection at the same time of the day can decrease preanalytical variation for AMH as it shows a diurnal variation (Bungum *et al*., 2011). In the study by Bungum et al., they found that AMH varied between 13-63% in patients with PCOS and between 10-230% in healthy controls within 24 hours (Bungum *et al*., 2011). This high intra-individual variation of AMH during the day can lead to exceeding clinical decision points and misinterpretation of test results by gynecologists. Additionally, drugs used for IVF treatment can cause false low and false high test results. It should be considered that GnRH agonists can cause elevated serum AMH concentrations in patients following *in vitro* fertilization treatment (Fu *et al*., 2021). Clinicians’ awareness of these preanalytical variables impacting test findings and the upcoming implementation of new standardized reference materials may lead to a more precise interpretation.
